# Poria cocos polysaccharide induced Th1-type immune responses to ovalbumin in mice

**DOI:** 10.1371/journal.pone.0245207

**Published:** 2021-01-07

**Authors:** Xiaoxiao Dong, Boye Li, Boyang Yu, Tian Chen, Qin Hu, Bo Peng, Wang Sheng

**Affiliations:** 1 The Faculty of Environment and Life, Beijing University of Technology, Beijing, PR China; 2 Institute of Chinese Materia Medica, China Academy of Chinese Medical Sciences, Beijing, PR China; Xinyang Normal University, CHINA

## Abstract

In the present study, we evaluated adjuvant potential of Poria cocos polysaccharide (PCP) on the Th1-type immune responses of C57/BL6 mice against ovalbumin (OVA). We first determined the effect of PCP on maturation of murine bone marrow derived dendritic cells (BMDCs), PCP significantly upregulated surface expression of MHCII, CD40, CD80, CD86 and enhanced production of IL-6 and IL-12p40. In addition, PCP affected receptor-mediated endocytosis, but not pinocytosis in BMDCs. Furthermore, OVA + PCP immunization induced specific cytotoxic CD8^+^ T cell killing of OVA (257–264) peptide pulsed cell. When mice were immunized subcutaneously in a week interval with OVA + PCP. Serum were collected for measuring OVA-specific antibody and splenocytes were harvested for analyzing CD69, IFN-γ ELISpot and cytokines production. The result indicated that OVA-specific IgG, IgG2a and IgG1 antibody levels in serum were significantly elevated by PCP compared with control. PCP increased OVA-specific IFN-γ-secreting CD8^+^, CD4^+^ T cells, promoted CD8^+^ T cell proliferation and up-regulated Th-1 type (IFN-γ, IL-2) cytokine production. In conclusion, data suggest that PCP enhanced cellular immune response and possess potential as a vaccine adjuvant for Th1 immune response.

## Introduction

For decades, immunoadjuvants are used as non-antigenic immune enhancer in vaccine to modify the immune response. Nowadays, most of the vaccines are developed using subunit antigen or recombinant proteins, those molecules are less immunogenic than live attenuated pathogens to elicit long-term immunity. Thus, adjuvants are in great need to boost immunogenicity [1]. Aluminum salts (hydroxide or phosphate) was registered for human vaccine since 1930s and had been the only adjuvant proved in clinic until recently. It’s still the most used adjuvant in current human vaccines. However, this adjuvant preferentially induces antibody response and shows weak or no T helper 1(Th1) or cytotoxic T cell response, which is essential for protection against many viruses or intracellular bacteria as well as cancerous cells. In addition, aluminum salts are often associated with local side effects, such as pain, redness and swelling at the injection site [2]. More recently, several adjuvants have been approved for human vaccines, including MF59, AS03, AS04, CpG ODN and AS01 [3,4], effectiveness and safety concerns remain challenges that hinder new adjuvant development, and new safe and potent adjuvants development are therefore needed.

Natural Polysaccharides are highly valuable macromolecules, characterized by intrinsic immune regulation, biocompatibility, biodegradability and low toxicity [5,6]. Their ability to boost effective cellular immunity, humoral immunity, mucosal immunity and excellent safety record making them promising candidate for novel adjuvant development. Chitosan and its derivatives, for example, are well documented as attractive alternative to alum [7,8].

Poria cocos is a type of fugus used as traditional medicine and functional food in East Asia for centuries. Poria cocos polysaccharide (PCP) is most abundance ingredient of Poria cocos and possess a variety of biological activities including immunomodulation, antitumor, anti-inflammation, anti-oxidation and hepatoprotection [9–12]. Intensive chemical analyses have identified that PCP are mainly composed of glucose, fucose, arabinose, xylose, mannose and galactose [13,14]. PCP was previously reported to improve immunogenicity against anthrax, rabies, Marburg virus, H1N1 in mice, dog and rhesus macaques models, primarily attributed to antigen-specific antibody response [15–18]. In the present study, we investigated the potential of PCP to enhance a T helper 1 (Th1)-mediated cellular immune response against ovalbumin (OVA) *in vitro* and *in vivo*.

## Materials and methods

### Materials

The single-stranded phosphonothioate ODN CpG 1668 (TCCATGACGTTC CTGATGCT) were synthesized by Sangon Biotech (Shanghai, China). OVA peptide SIINFEKL (OVA peptide 257–264) was purchased from Chinese Peptide Company (Nanjing, China), OVA were obtained from Sigma (Sigma, USA). PCP was purchased from YuanYe Company (Shanghai, China). The characterization of PCP was described in S1 Fig. PCP was found to mainly consist of mannose, glucose, galactose, fucose at a molar ratio of 1.15: 2.42: 1.88: 1.0. The Number-average molecular weight (Mn), mass average molar mass (Mw) and Z-average Molecular Weight (Mz) of PCP were 1.28 × 10^4^, 2.06 × 10^4^, and 2.70 × 10^5^ g/mol, respectively (S2 Fig). The polydispersity coefficients were 1.60 (Mw/Mn) and 21.06 (Mz/Mn). All materials for vaccine were purified by Pierce™ High Capacity Endotoxin Removal Spin Columns Thermo Fisher Scientific, USA) to remove endotoxins and the endotoxin levels were determined to be consistently below 5 EU/mL by ToxinSensor^TM^ Endpoint Chromosome Endotoxin Detection Kit (Genscript, USA).

### Mice

Female C57BL/6 mice (18–22 g, 6–8 weeks, n = 115) were purchased from Beijing Vital River Laboratory Animal Technology Co., Ltd (Beijing, China) and were acclimated to the laboratory housing for 3 to 4 days. Mice were housed in a cage of three in specific-pathogen-free (SPF) conditions with temperature of 18–22°C, humidity of 40–60% and a 12:12 light/dark cycle. All animals had free access to sterile food and water. Animals were monitored daily for health status and weighed once a week. Procedures were performed by experienced operators and involved only brief restraint. After injection procedures, animals were closely monitored for signs of pain or distress, motility and level of food consumption. Animals that reached the humane endpoints will be euthanized. No death or adverse effect was observed in mice during the study. At the end of experiments, mice are euthanized by cervical dislocation. The researchers were blinded with regards to pharmacological treatment while processing data. All experiments were approved by the Institutional Animal Treatment and Use Committee of Institute of Basic Research in Clinical Medicine, China Academy of Chinese Medica Sciences (code: 20190901058) and conducted in accordance with Chinese National Guidelines (GB/T 35892–20181). The ARRIVE Essential 10 checklist were included in S1 Checklist.

### Cells culture

Mouse bone marrow-derived DC (BMDC) were generated from bone marrows cell of female C57BL/6 mice as previously described [19]. Briefly, the fresh BMDCs were extracted from the femur and tibia bones of 6–8 weeks female C57BL/6 mice. Cells with cultured in the presence of 20 ng/mL GM-CSF (PeproTech, US) in RPMI 1640 medium supplemented with 2 mM glutamine, 100 U/ml penicillin, 100 mg/ml streptomycin, 50 μM 2-mercaptoethanol (Invitrogen, US) and 10% heat-inactivated fetal calf serum (FBS, Gibco, US). Non-adherent cells were collected from culture as immature BMDCs at day 6 for subsequent experiments. DC2.4 mouse dendritic cells were purchased from Bena Culture Collection (BNCC, China) and were cultured in RPMI 1640 medium supplemented with 2 mM glutamine, 100 U/ml penicillin, 100 mg/ml streptomycin, 50 μM 2-mercaptoethanol and 10% heat-inactivated FBS.

### Cell viability

DC2.4 cells were treated with increasing concentration of PCP ranging from 25 μg/mL to 1600 μg/mL. Cellular viability was assessed using Cell Counting Kit-8 (Dojindo, Japan) at 24, 48 and 72 hours after treatment. The relative cell viability was calculated as a percentage of untreated control cells.

### Flow cytometry analysis

Flow cytometry analysis was performed using BD FACS Calibur^TM^ flow cytometer (BD Bioscience) and analyzed using FlowJo software V10 (Tree star). For cell surface staining, cells were washed with PBS and incubated with anti-mouse CD16/32 antibody for 15 min, followed by staining with antibody cocktail in PBS + 1% FBS for 1 h. Cells were then collected by 3 times washing of PBS and resuspended in PBS + 1% FBS for FACS analysis.

### Uptake assays

PCP was labeled with fluorescein isothiocyanate (FITC) via tyramine reduction method as previously reported [20]. For uptake assay, DC2.4 cells were seeded in 96-well plates and incubated PCP-FITC at 200, 400 and 800 μg/mL, BMDCs were cultured with PCP-FITC at 50, 100, 200 μg/mL. After 30 min, cells were harvested and stained with anti-mouse CD11c antibody before FACS analysis. For uptake competition assay, BMDCs were pre-incubated with 200 μg/mL Fucoidan (Solarbio, China), 20 μg/mL anti-mouse Dectin-1 antibody (InVivoGen, US), 200 μg/mL Laminarin (InVivoGen, US), 200 μg/mL D-Galactose (Aladdin Bio-Chem Technology, China) or 200 μg/mL Mannan (Solarbio, China). After 30 min, BMDCs were cultured with PCP-FITC (100 μg/mL) for 45 min before FACS analysis. For antigen uptake, BMDCs were first incubated with PCP (200 μg/mL) for 30 min, then DCs were co-cultured with OVA-atto 488 (5 μg/ml), Dextran-FITC (MW 40,000, 100 μg/ml) or Lucifer yellow VS dilithium salt (LY, 100 μg/ml) for 45 min before analysis of antigen uptake by CD11c^+^ BMDCs.

### BMDC activation

Immature BMDCs were counted and plated at 1 ×10^6^ cells per well in the 24-well plate and treated with PCP, CpG as positive control or PBS as negative control. After 24 h, cells were collected and stained with FITC anti-mouse CD11c, PE anti-mouse MHC-II, APC anti-mouse CD80, PerCP/Cyanine5.5 anti-mouse CD86 and PerCP/Cyanine5.5 anti-mouse CD40 (BioLegend, US) for 1 h prior to flow cytometry. The supernatant of BMDC culture were also harvested to measure the cytokine concentration of IL-6 and IL-12 using Mouse IL-6 ELISA MAX Deluxe and Mouse IL-12 p40 ELISA MAX Deluxe kits (BioLegend, US) as detailed in the manufacturer’s instruction.

### In vivo killing assay

On day 0, female C57BL/6 mice were randomly divided into 4 experiment groups (n = 24) and one control group (n = 6). Each mouse in experiment group was immunized subcutaneously with 10 μg OVA protein and PCP at a dose of 250 μg, 500 μg, 1 mg or 10 μg CpG, control mice were treated with PBS. Six days later, mice were injected intravenously with target cells consisting of a 1:1 mixture of SIINFEKL peptide-pulsed splenocytes labeled with 0.5 μM carboxyfluorescein succinyl ester (CFSE) and un-pulsed splenocytes labeled with 5 μM CFSE. After 24 h, mice splenocyte were harvested by Collagenase IV/DNase I (Sigma, US) digestion, followed by flow cytometry analysis to determine the antigen-specific killing using the formula: 100-[100 x (%CFSE_peptide-vaccinated_/%CFSE_no peptide-vaccinated_)/(%CFSE_peptide-PBS_/%CFSE_no peptid_e_-PBS_)].

### Immunization of mice

Female C57BL/6 mice were randomly divided into 4 groups (n = 24). Mice were immunized subcutaneously on day 0, day 7 and day 14 with PBS (control group, n = 6), OVA (10 μg per mouse, n = 6), OVA in combination with PCP (500 μg per mouse, n = 6) or OVA with CpG ODN (10 μg per mouse, n = 6). On day 21, mice were anesthetized with isoflurane and sacrificed after serum and spleens were harvested for immunological analysis.

### ELISPOT

Mouse IFN-γ ELISPOT assay (Dakewe, China) were conducted according to the manufacturer’s instruction. Briefly, splenocytes were harvested and seeded at 2× 10^5^ cells/well in pre-coated 96 well plate, cells were then activated of 1 μg/mL OVA peptide 257–264 (SIINFEKL) or 300 μg/mL OVA protein per well for 36 h, PMA/ionomycin was used as a positive control. The number of spots on ELISPOT plates were counted automatically. Results are shown as spot-forming cells (SFCs) per 10^5^ splenocytes.

### Measurement of OVA antibody response and cytokine release by ELISA

Blood was collected on day 21 from immunized mice and serum was separated by centrifugation. OVA-specific IgG, IgG1 and IgG2a in serum were quantified by IgG Mouse ELISA kit (Thermo Fisher Scientific, US) following the manufacturer’s instruction with slight modifications. In brief, Corning® 96-well Flat Bottom Polystyrene High Bind Microplates (Corning, US) were coated with 200 μg/mL OVA overnight at 4°C and blocked with blocking buffer for 2 h at room temperature, followed by incubation with serum (diluted 1:1200 to detect IgG and 1:500 to detect IgG1, IgG2a). Plates were washed for 5 times and incubated with HRP-conjugated anti-mouse IgG (Thermo, US), IgG1 and IgG2a (Bethyl, US). Subsequently, plates were washes and incubated with TMB solution, the reaction was stopped by sulfuric acid and the absorbance were measured at 450 nm.

Splenocytes were harvested on Day 21 and seeded in 2 x10^6^ cells/mL with OVA (300 μg/mL) or OVA peptide 257–264 (1 μg/mL). After 48 h incubation, CD69 expression was analyzed using FACS analysis. Supernatant were collected at 72 h and measured for IL-4, IL-2, IL-17 and IFN-γ concentration using ELISA kits (BioLegend, US) according to the protocol supplied by the manufacture.

### Statistical analysis

Results were expressed as means ± standard deviations (SD). Statistical analysis was performed by a One-way ANOVA using the GraphPad Prism software 7. Differences between groups were considered significant at a p-value < 0.05. The animal sample sizes were calculated based on pilot study data using InVivoStat software’s (version 3.7, UK) Power Analysis with the minimum level of α of 0.05 and statistical power of 80%.

## Results

### PCP induced maturation of BMDCs

**Fig 1:**
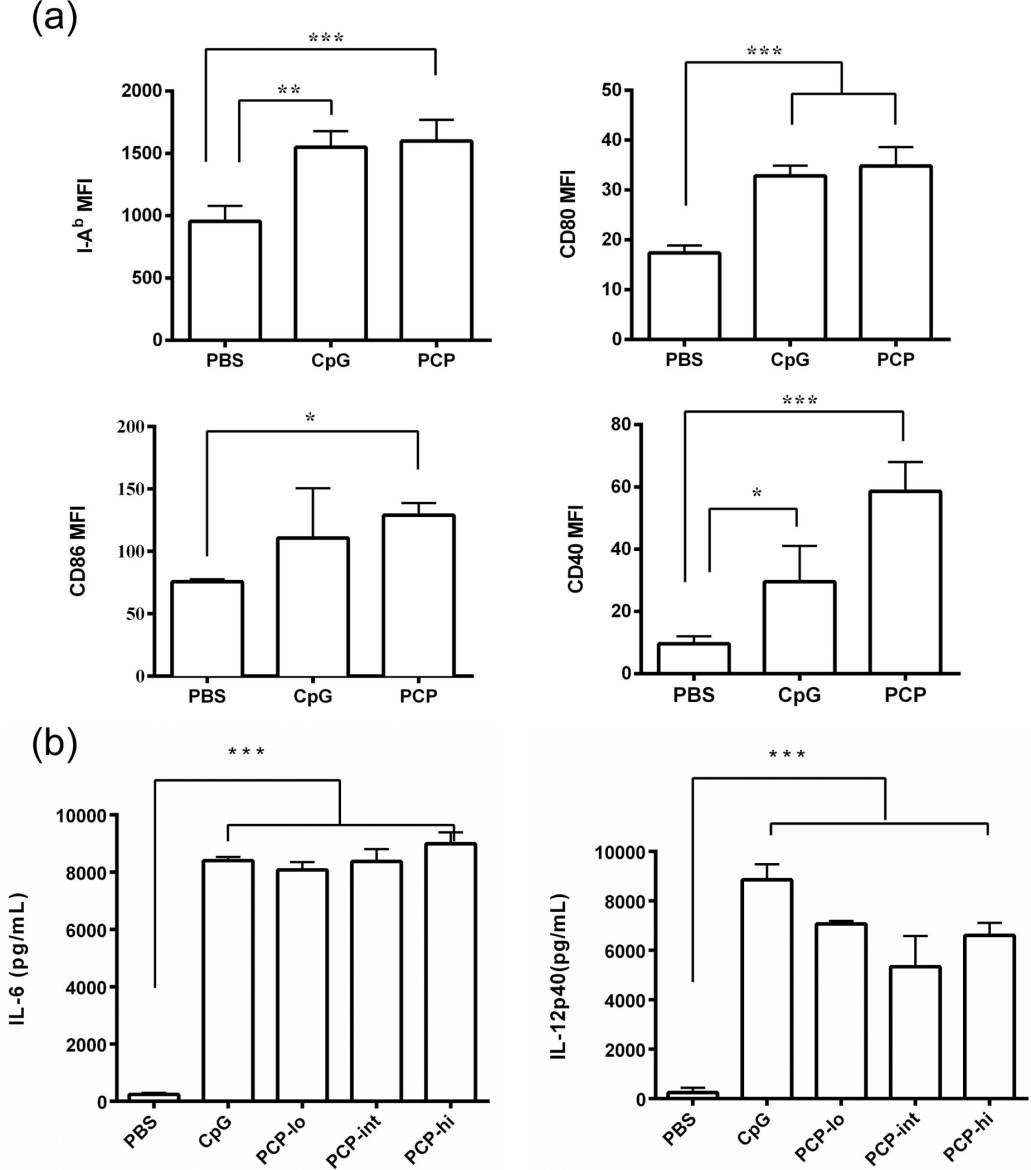
PCP induced BMDC maturation *in vitro*. Immature BMDCs were isolated from C57BL/6 female mice and stimulated by 10 to 100 μg/mL PCP or 10 μg/mL CpG for 24 h. (a) The cell surface expression of I-A^b^, CD80, CD40 and CD86 were analyzed by FACS. (b) Supernatant of BMDC culture were collected, IL-6 and IL-12p40 production was measured by ELISA assay. The data shown are representative of at least 4 independent experiments. Data are expressed as the mean ± SD, *, P ≤ 0.05, **, P ≤ 0.01, ***, P≤ 0.001 vs PBS control.

We first analyzed the effect of PCP on BMDC maturation, BMDCs were harvested from female C57BL/6 mice and cultured with GM-CSF for 6 days, immature BMDCs were then co-cultured with 100 μg/ml PCP or with 10 μg/ml CpG as a positive control. PCP induced 2-fold increase in MHC-II expression and 2-3-fold up-regulation in surface expression of co-stimulatory molecules including CD40, CD80 and CD86 (Fig 1A). Furthermore, the supernatant of BMDC culture were measured for IL-6 and IL-12p40 (Fig 1B). The secretion of IL-6 was found dose-dependently elevated after 24 h treatment of PCP ranging from 10 to 100 μg/ml PCP. All three doses of PCP also increased the production of IL-12, which is a potent stimulator of Th-1 immunity. The data indicated that PCP was able to promote *in vitro* maturation of BMDCs.

### PCP induced OVA specific cytotoxic CD8^+^ T cell killing

**Fig 2:**
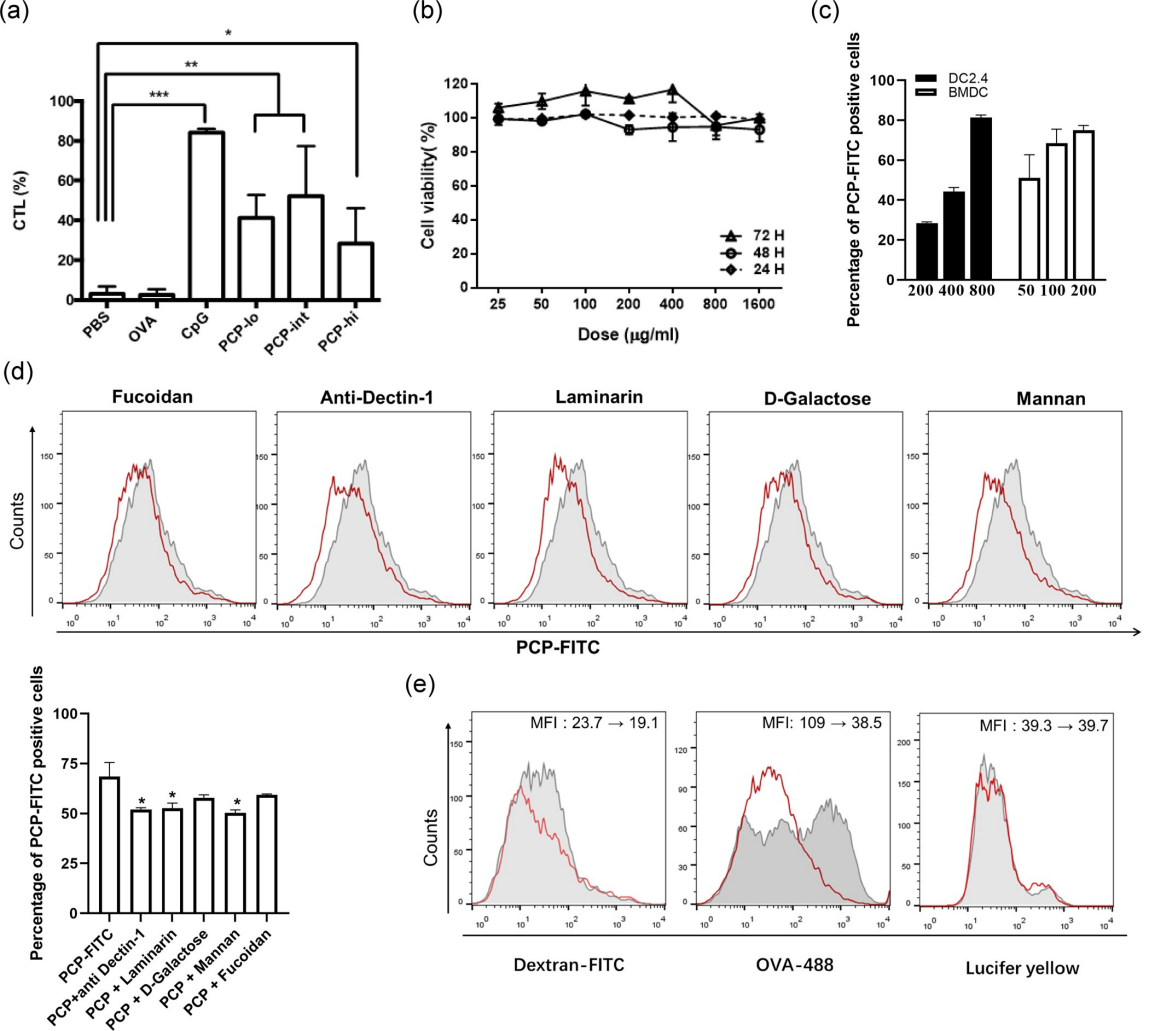
Cytotoxicity, uptake and enhanced CTL killing of PCP. (a) Mice were immunized s.c. with 10 μg OVA mixed with CpG (10 μg) or different concentrations of PCP (250 μg, 500 μg and 1 mg per mouse). On day 6, target cells were pulsed with or without OVA peptide (257–264) and stained with low or high concentrations of CFSE, two populations were mixed at a ratio of 1:1 and injected i.v. to immunized mice. After 24 h, splenocytes were harvested and analyzed by FACS analysis. The percentage of OVA specific killing were calculated and expressed as mean ± SD. (b) DC2.4 cells were incubated with PCP ranging from 0 to 1.6 mg/mL, cell viability was evaluated by CCK-8 assay at 24 h, 48 h and 72 h. (c) DC2.4 cells were incubated PCP-FITC at 200, 400 and 800 μg/mL, BMDCs were incubated PCP-FITC at 50, 100 and 200 μg/mL. Cells were harvested and analyzed by FACS. Data are expressed as percentage of FITC positive cells. (d) BMDCs were pre-incubated with fucoidan (200 μg/mL), anti-mouse Dectin-1 antibody (20 μg/mL), laminarin (200 μg/mL), D-Galactose (200 μg/mL) or mannan (200 μg/mL) for 30 min before PCP-FITC incubation, cells were harvested for FACS analysis. The histograms represent PCP uptake by untreated cells (grey area), and cells pre-incubated with multiple inhibitors (red line). The bar graph shows the percentage of PCP-FITC positive cells. (e) BMDCs were co-cultured with OVA-Atto 488 (5 μg/ml), Dextran-FITC (MW 40,000, 100 μg/ml) or Lucifer yellow VS dilithium salt (LY, 100 μg/ml) after pre-incubation with PCP (100 μg/mL). The uptake of OVA, Dextran and LY were shown in histograms (grey area: no pre-incubation with PCP, red line: cells incubated with PCP). All data are representative of 2–3 independent experiments. *, P ≤ 0.05, **, P≤ 0.01, ***, P≤ 0.001 vs PBS control.

To investigate whether PCP had a direct effect of promoting antigen-specific cytotoxic T cell response *in vivo*, mice were immunized with OVA or OVA in combination with PCP. A week later, mice were administrated i.v. with CFSE labeled splenocytes loaded with OVA peptide (257–264) and untreated splenocytes mixed at 1:1 ratio. PCP induced significant cytotoxic effect against splenocytes pulsed with OVA peptide (257–264) peptide with an average killing of 41.4% (250 μg), 52.2% (500 μg) and 28.5% (1 mg), respectively. Immunizing with CpG induced a strong CTL response with 84.23 + 1.48% of peptide-pulsed target cells being killed, whereas OVA alone, as a negative control, gave an average killing of 2.54% target cells (Fig 2A). The data indicated that PCP induced antigen-specific killing activity of CD8^+^ T cells *in vivo* and 500 μg of PCP per mouse was used for the following *in vivo* tests. In addition, the lymphocyte proliferation assay was conducted to assess the unspecific stimulation of lymphocytes after PCP treatment. As expected, PMA/ionomycin or CpG induced unspecific proliferation of mice splenocytes and production of IL-2. PCP had no effect on splenocytes proliferation but promoted IL-2 secretion at 50 μg/mL (S3 Fig).

Furthermore, DC2.4 cells were exposed to a range (25–1600 μg/mL) of PCP for 24, 48 and 72 h, there was no observable cytotoxic effect at all tested time points (Fig 2B). The data suggested the low cytotoxicity of PCP *in vitro*. To detect the uptake of PCP-FITC *in vitro*, DC2.4 cells and BMDCs were incubated with an increasing concentration of PCP-FITC, and the increase of FITC positive cells were observed in both DC2.4 cells and BMDCs (Fig 2C), demonstrating that PCP uptake is dose dependent. To further clarify the uptake route of PCP-FITC in BMDCs, we pre-incubated cells with fucoidan, anti-mouse Dectin-1 antibody, laminarin, mannan and D-galactose, which were the main components of PCP. Although they all affected the uptake of PCP-FITC, only anti-mouse Dectin-1, laminarin and mannan induced significant reduction on the percentage of PCP-positive cells (Fig 2D). The data indicated that multiple receptors were involved in the internalization of PCP. To further test whether PCP interfere with receptor-mediated endocytosis or pinocytosis of BMDCs, we incubated BMDCs with OVA-Atto 488, Dextran-FITC or LY (uptake exclusively by pinocytosis), the data showed that PCP blocked the uptake of OVA and dextran at different levels, however, no reduction in uptake of LY was observed (Fig 2E), indicating that PCP interfered with receptor-mediated endocytosis and did not interfere with pinocytosis.

### PCP induced potent cellular immune response against OVA antigen

**Fig 3:**
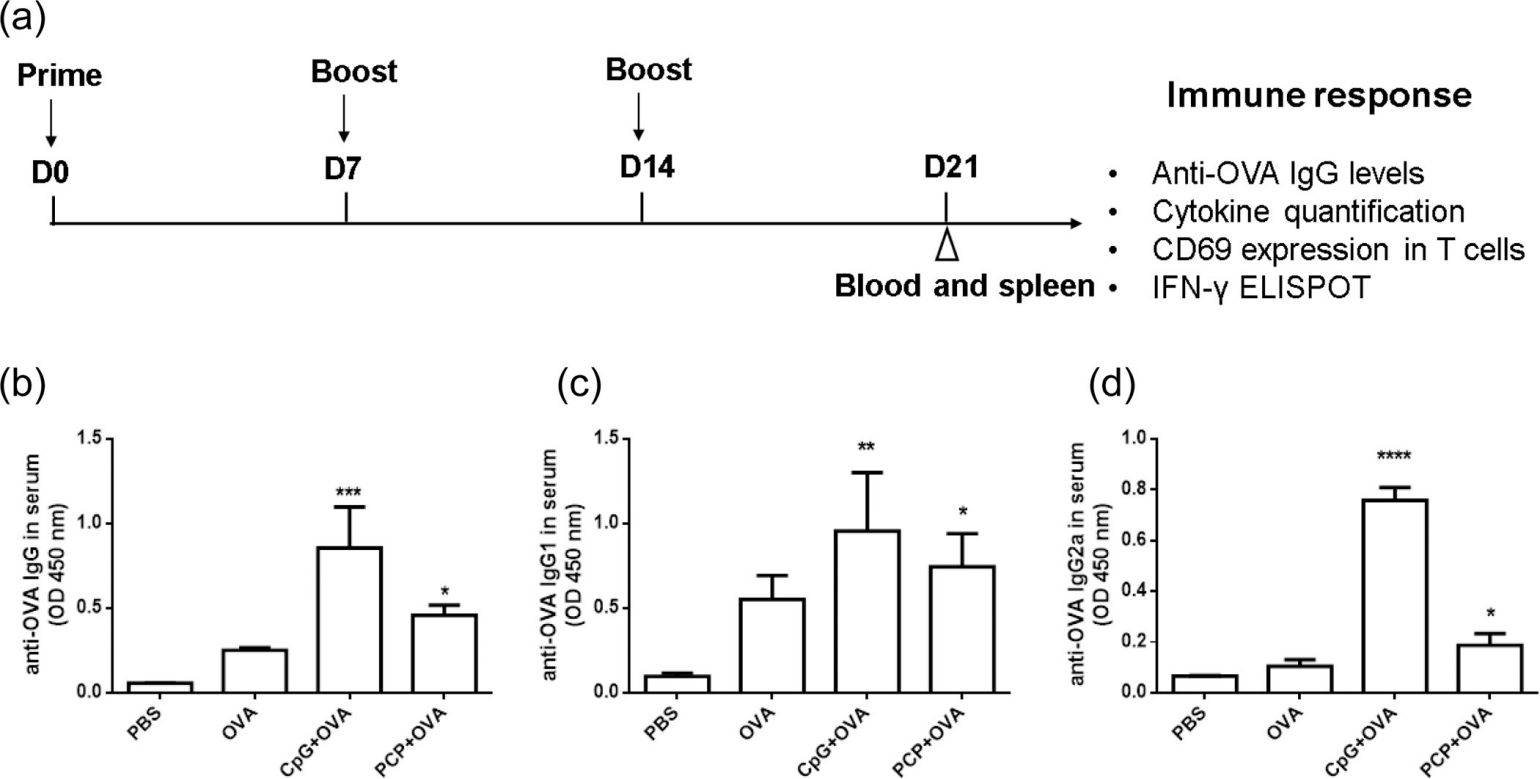
PCP induced OVA specific IgG antibody production. (a) Timeline representation of immunization schedule. Mice were immunized 3 times (s.c.) with 10 μg OVA mixed with CpG (10 μg) or PCP (500 μg) with oneweek intervals between prime and boost. Blood and spleens were collected at day 21. OVA-specific IgG (b), IgG1 (c) and IgG2a (d) antibodies in serum were assayed by ELISA assay. The graphs show absorbance at 450 nm (OD 450 nm). *, P ≤ 0.05, **, P ≤ 0.01, ***, P≤ 0.001, ****, P≤ 0.0001 vs control.

Mice received 3 vaccinations 1 week apart with 500 μg PCP or 10 μg CpG per mouse. Blood was collected and assayed for anti-OVA IgG by ELISA assay. Mice spleen were harvested and examined for the frequency of IFN-γ–producing cells, CD69 positive cells and cytokine production (Fig 3A). Serum IgG analysis showed that after 3 immunizations, PCP and CpG resulted in significant increase of serum anti-OVA IgG (Fig 3B), IgG1 (Fig 3C) and IgG2a (Fig 3D) than control group.

**Fig 4:**
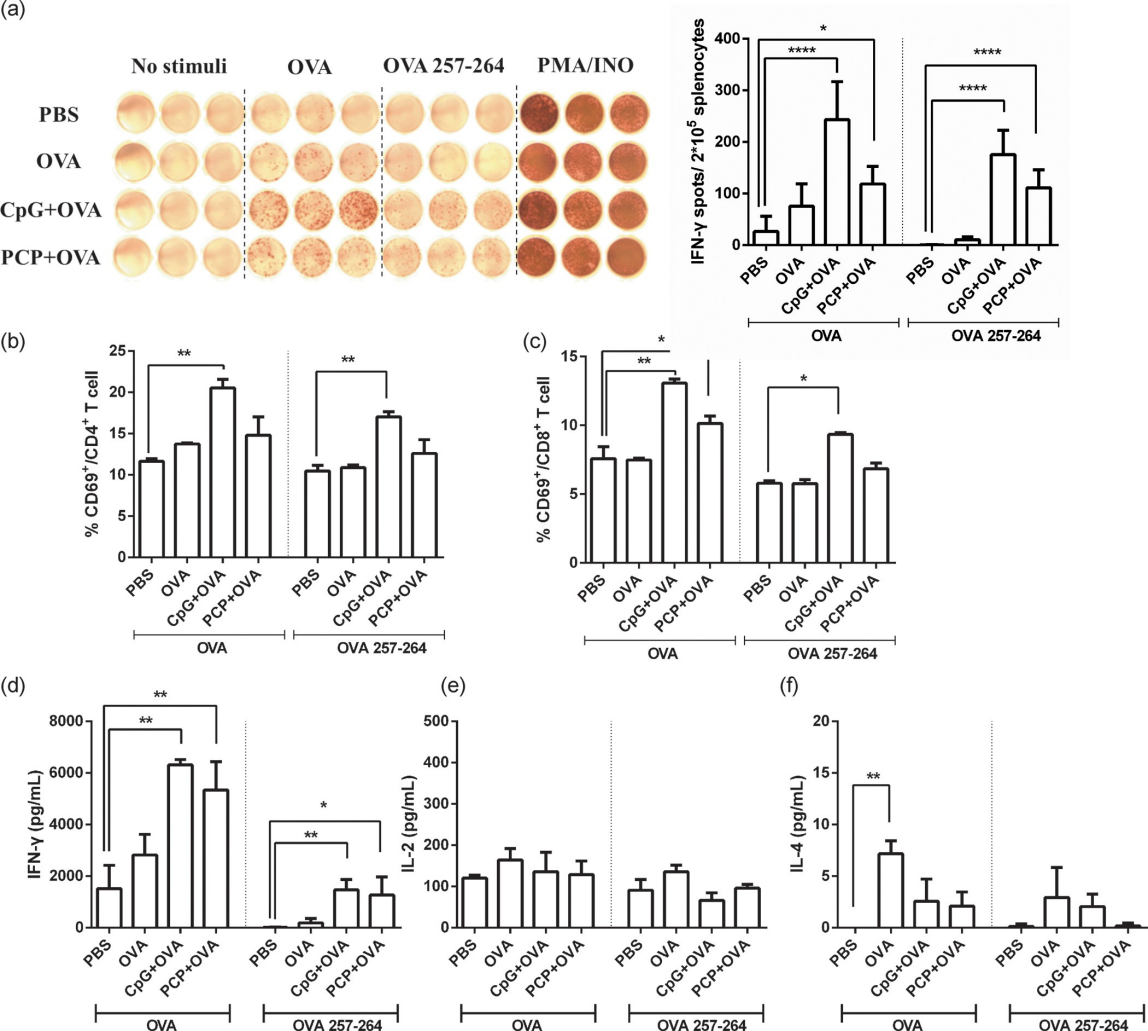
PCP enhanced OVA specific T cell response. Mouse spleens were collected on day 21. Splenocytes were co-cultured with OVA (300 μg/mL) or OVA peptide (257–264) (1 μg/mL), PMA/ionomycin was used as a positive control. (a) After 36 h, the IFN-γ secreting cells were visualized and analyzed by ELISPOT. The number of OVA stimulated and OVA peptide (257–264) stimulated IFN-γ secreting spots was measured and calculated as IFN-γ spot-forming cells/ 2 x 10^5^ cells. After 48 h incubation, CD69 positive CD4 T cells (b) and CD8 T cells (c) were analyzed by FACS analysis. After 72 h, the supernatant was harvested and the production of IFN-γ (d), IL-2 (e) and IL-4 (f) in culture were assayed by ELISA. Data are expressed as the mean ± SD, *, P ≤ 0.05, **, P ≤ 0.01, ****, P ≤ 0.0001 vs PBS control.

T cell response were determined by ex vivo re-stimulation of splenocytes with OVA antigens and measurement of CD8^+^ and CD4^+^ T cells producing IFN-γ against OVA peptide (257–264) and OVA protein, respectively (Fig 4A). IFN-γ ELISpot assay showed that PCP significantly induced OVA specific IFN-γ secreting T cell and OVA peptide (257–264) specific IFN-γ secreting CD8^+^ T cell. The proliferation of CD8^+^ T cells after OVA stimulation was confirmed by CD69 staining (Fig 4B and 4C). T cell immunity were also assessed by analysis of OVA-specific cytokine expression in splenocytes. The different subset of cytokines gives an indication of a Th2 or Th1 bias responses, respectively. Immunization with PCP elevated IFN-γ cytokines in comparison with control mice (Fig 4D and 4E), whereas failed to produce IL-4 cytokines (Fig 4F). In addition, there was no detectable IL-17 in supernatant of splenocytes culture. Together, the data indicated an adjuvant effect of PCP with enhanced antibody response, IFN-γ secreting and antigen specific CD8^+^ T cell activation.

## Discussion

In the present paper, we investigated the ability of PCP to enhance cellular immune response against OVA antigen. PCP induced potent activation of BMDC, as evidenced by surface molecule (CD80, CD86, CD40 and MHC class II) upregulation as well as IL-6 and IL-12p40 secretion. In addition, no cell cytotoxicity was detected with a wide range of PCP. SR-A and Dectin-1 receptor were involved in PCP uptake by DCs. PCP, when co-injected with OVA, led to the production of OVA-specific IgG antibody and IFN-γsecreting CD8^+^ and CD4 T^+^ cells. The cross-presentation of OVA antigen to OVA- specific CD8^+^ T cells was confirmed by antigen specific killing of cytotoxic CD8^+^ T cells. Thus, PCP potently elicited a strong cellular immune response.

Adjuvants are widely used in vaccine preparation to increase the immunogenicity of weak antigens. Apart from safety and efficacy issues, other key aspects are need to take into account in the choice of adjuvant, such as the capability to induce antigen cross-presentation in DCs [21]. For vaccines against intracellular antigens (virus, intracellular microbes) and cancer vaccines, efficient cross-presentation of antigen by DCs is pivotal for initiating optimal CD8^+^ T cell response. PCP, extracted from Poria cocos sclerotium, has been extensively studied as an immunomodulating agent [22–25]. According to previous reports, PCP significantly stimulated antibody responses, enhanced cytokines secretion and exerted protective role against MARV, rabies, and H1N1 [15,16,18]. To further elucidate that capacity of PCP to induce antigen-cross presentation and enhance cellular immune response, we used OVA as a model antigen in the present study. The MHCI epitope (OVA 257–264) and MHCII epitope (OVA 323–339) of OVA protein were well characterized and extensively used to study MHCI and MHCII antigen presentation and cross-presentation pathways [26,27]. TLR9 ligand CpG ODN is a well-documented Th1 biasing adjuvant that aids cross-presentation of MHC class I restricted antigen and has been approved for clinical use in Heplisav-B (HepB-CpG) vaccine since 2018 [28,29], we used CpG ODN 1668 as a positive control in experiments.

Efficient presentation and cross-presentation occur upon induction of DC maturation. When DCs sense the presence of potential pathogen via PAMPs or other signals, immature DCs will upregulate the cell surface expression of MHC-II and co-stimulatory molecules like CD80 and CD86 to transform into mature cells. DCs in full maturation status also secreted assorted cytokines like IL-6, TNF-alpha and in particular IL-12 which induces the differentiation of T cells to Th1 cells that produce IFN-γ [30,31]. Our data showed that PCP induced the maturation of DCs via increasing MHC class II and costimulatory molecule expression and cytokine secretion. In addition, no cell cytotoxicity was detected. To clarify the route of uptake of PCP into DC cells, we used multiple sugars, which are the main components of PCP to block the uptake of PCP into BMDCs. The data showed that they all affected PCP uptake, but only laminarin, mannan and anti-mouse Dectin-1 exhibited significant inhibition. According to previous reports, Laminarin is able to block β-glucan specific pattern recognition Dectin-1 receptor and mannan is widely used to saturate mannose receptors on DCs [32–34]. The data indicated PCP might use a range of receptors (Dectin-1, mannose receptor, etc.) for internalization. We further found that PCP interfered with OVA and dextran uptake into DCs, OVA and dextran could be uptake via receptor mediated endocytosis (mannose receptor, SR-A, Dectin-1, etc.) and pinocytosis. The uptake of LY was not affected indicating that PCP mainly affected receptor-mediated endocytosis but not pinocytosis. To further investigate whether mature DCs activated by PCP could cross-prime peptide-specific CD8^+^ cytotoxic T cells, we co-injected C57/BL6 mice with PCP and OVA. Before vaccination, OVA-specific CD8 T^+^ cells were supposed to present at very low level in the T cell repertoire. If PCP could enhance the DC cross-presentation of exogenous OVA protein, the CD8^+^ T cells, which specifically recognize the SIINFEKL (MHC-class I-restricted) epitope of OVA will be primed and able to kill target cells pulsed with SIINFEKL peptide. The hypothesis was confirmed when PCP was administrated and resulted in over 50% of killing. What’s more, the splenocytes were harvested from immunized mice and ex vivo re-stimulated with OVA or OVA peptide (257–264) for IFN-γ ELISpot assay, the data indicated that PCP were potent at cross-priming and boosting anti-OVA cytotoxic CD8^+^ T cells. The activation of cytotoxic CD8 T^+^ cells were further confirmed by enhanced production of Th1 type cytokines in ELISA analysis. Also, consisting with previous report, PCP was able to promote antigen-specific IgG, IgG1 and IgG2a production [15,18], suggesting the capacity of PCP to enhance both cellular and antibody immune response.

Collectively, these data suggested that PCP stimulated DC maturation, cytokine secretion, antigen presentation, cross-presentation and promoted Th1-dominant response. PCP has great potential to be used as candidate adjuvant capable of driving cell-mediated immunity. Further research is warranted to investigate the antigen uptake and presentation pathway involved in PCP stimulated immune response.

## Supporting information

S1 Checklist ARRIVE essential 10 checklist. (PDF)

S1 Fig The FT-IR spectrum of PCP. (TIF)

S2 Fig The GPC profile of PCP using light scattering (LS) and differential refractive index (dRI). (TIF)

S3 Fig The effects of PCP on unspecific stimulation of splenocytes. After 48 h incubation, the splenocytes proliferation was detected using CCK-8 assay (a) and IL-2 production from supernatant was detected using ELISA (b). Data are expressed as the mean ± SD, ***, P ≤ 0.001, ****, P ≤ 0.0001 vs PBS control.
(TIF)

S1 File Supplementary materials and methods. (DOCX)
