# Supplementary figures and images for: Poria cocos polysaccharide induced Th1-type immune responses to ovalbumin in mice
Source: PLoS One. 2021 Jan 7;16(1):e0245207. doi: 10.1371/journal.pone.0245207 (PMC7790389; doi:10.1371/journal.pone.0245207)

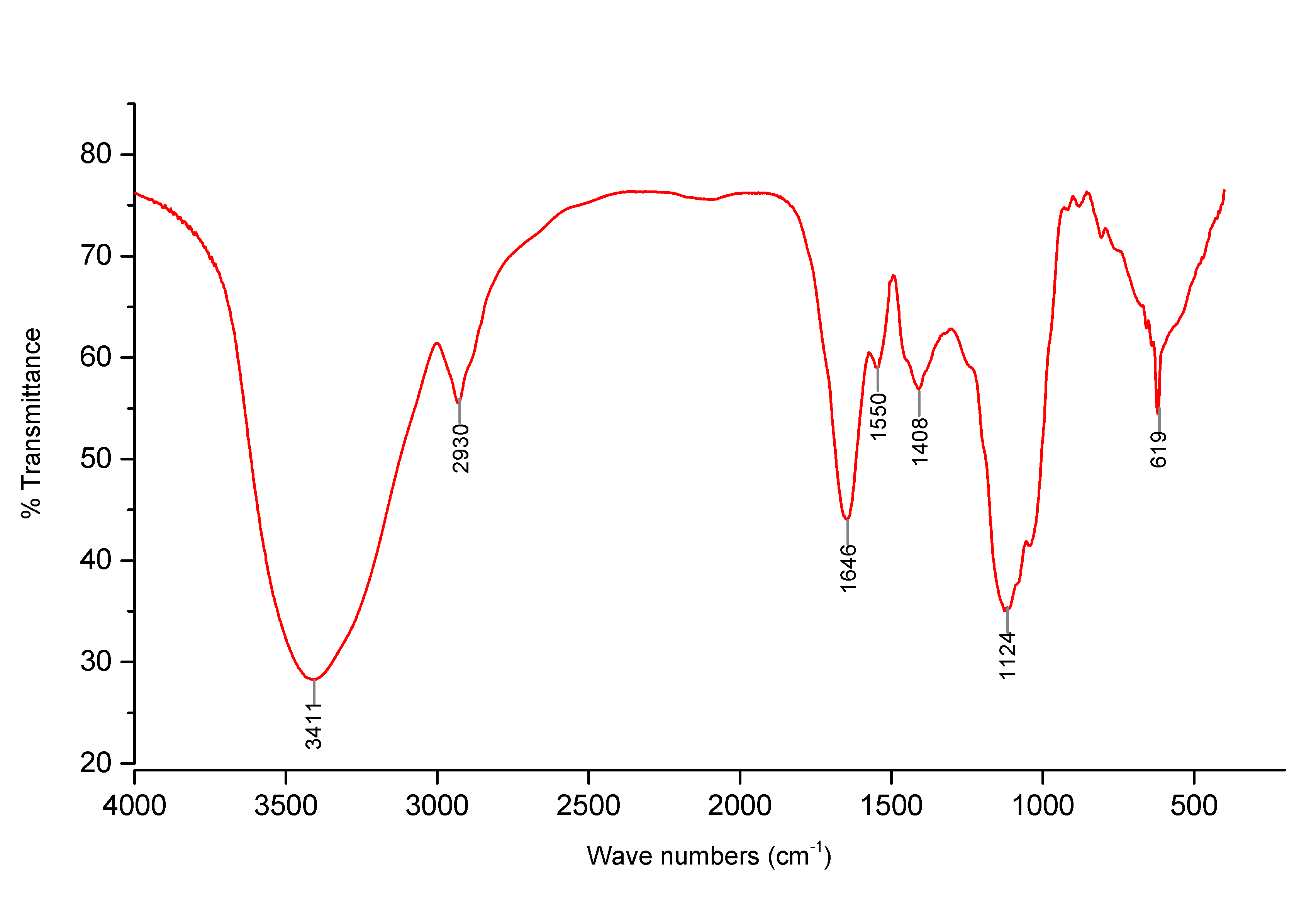

Supplement: S1 Fig — (TIF) [file pone.0245207.s002.tif]

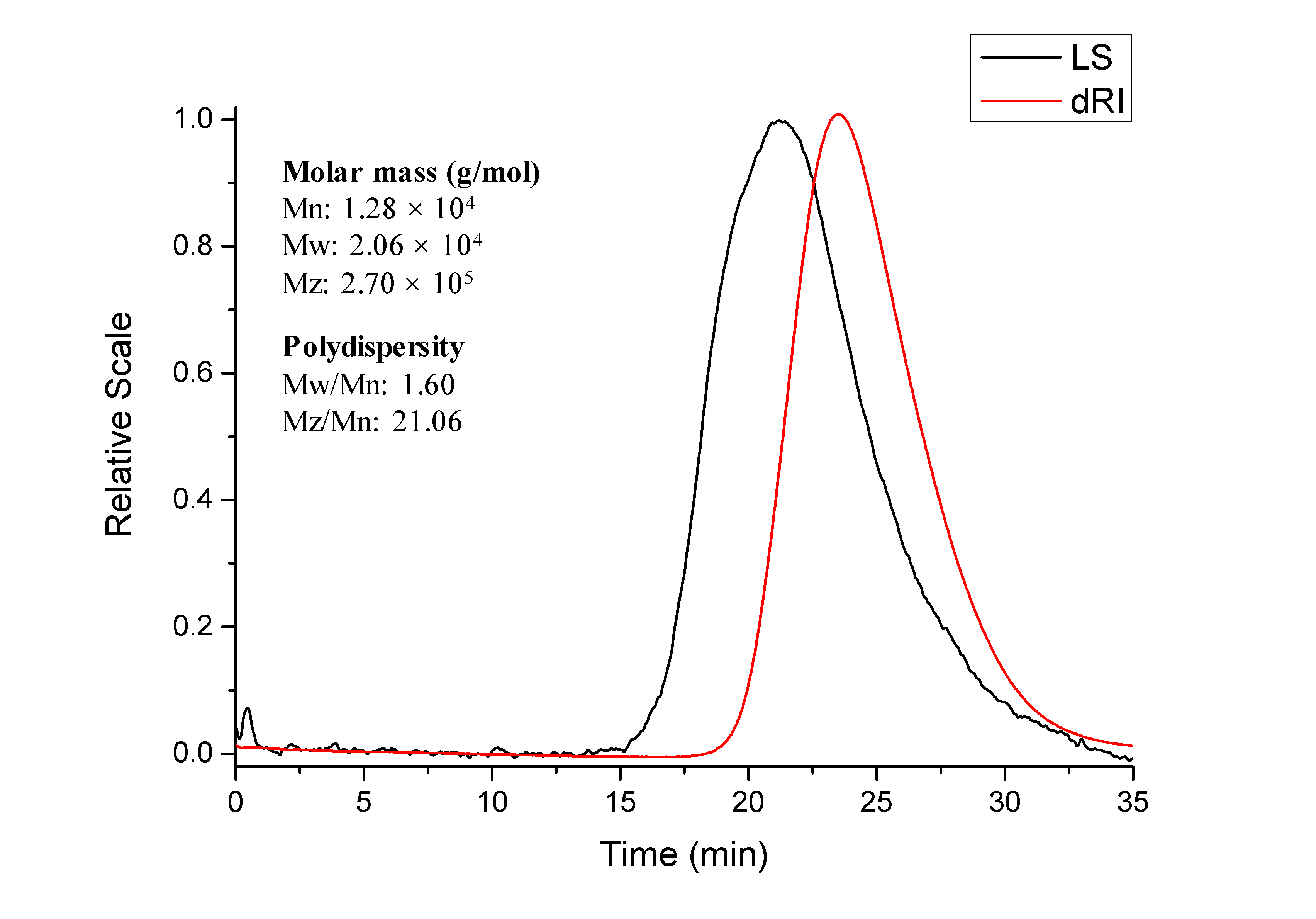

Supplement: S2 Fig — (TIF) [file pone.0245207.s003.tif]

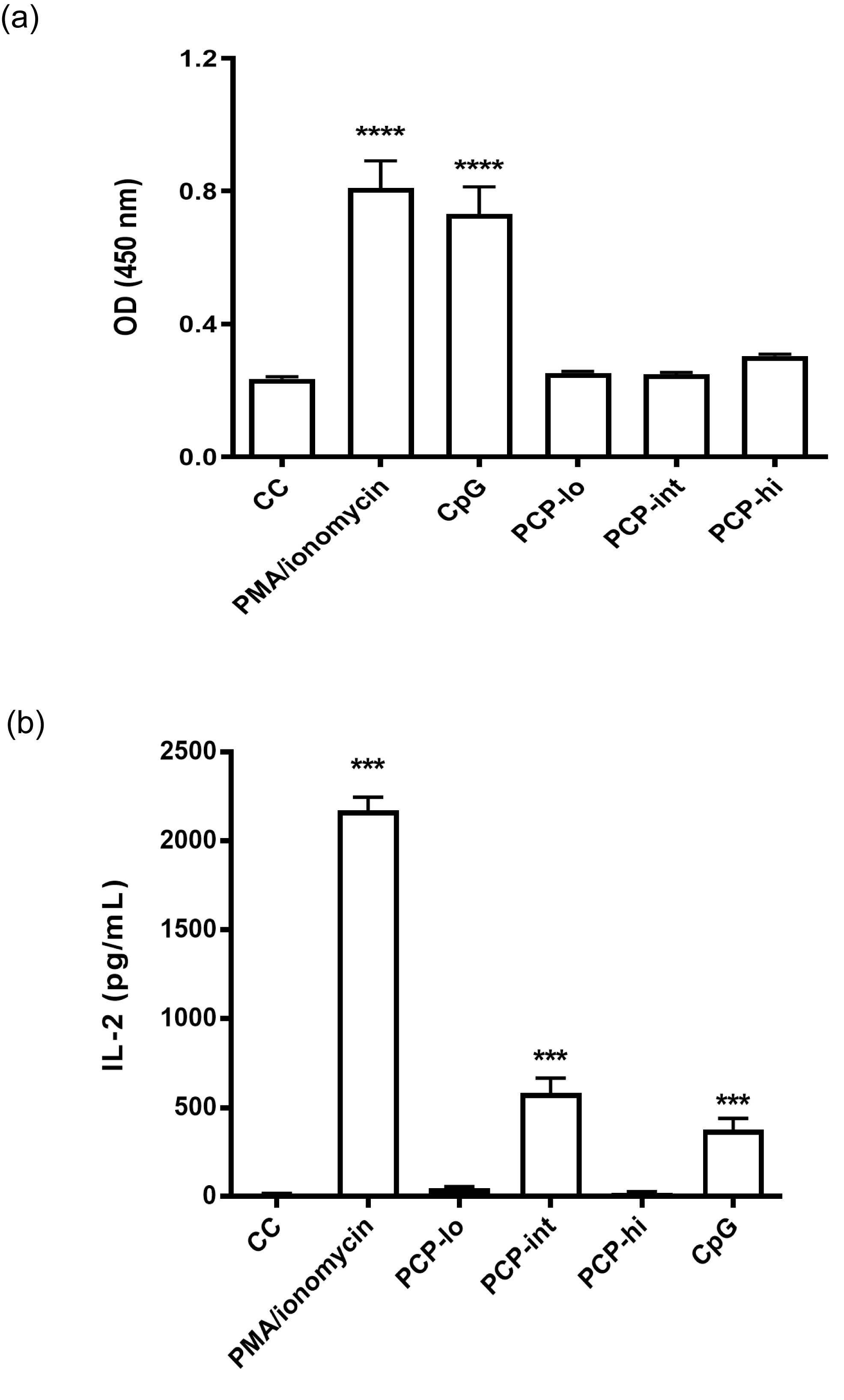

Supplement: S3 Fig — After 48 h incubation, the splenocytes proliferation was detected using CCK-8 assay (a) and IL-2 production from supernatant was detected using ELISA (b). Data are expressed as the mean ± SD, ***, P ≤ 0.001, ****, P ≤ 0.0001 vs PBS control. (TIF) [file pone.0245207.s004.tif]
