# Supplementary material for: Poria cocos polysaccharide induced Th1-type immune responses to ovalbumin in mice
Source: PLoS One. 2021 Jan 7;16(1):e0245207. doi: 10.1371/journal.pone.0245207 (PMC7790389; doi:10.1371/journal.pone.0245207)
Supplement: S1 File — (DOCX) [file pone.0245207.s005.docx]

**Supplementary Data**

**Materials and Methods**

Characterization of PCP

The monosaccharide composition of PCP was assayed using Agilent 1200 HPLC system (Agilent Technologies, US) as previously described [1]. The molar mass and size distribution were analyzed on a gel permeation chromatography (GPC) using a Wyatt GPC/SEC-MALS system (Wyatt Technology) with Dawn HELEOS-II MALS detector, Optilab rEX detector and OHpak SB-806 HQ column. For infrared spectral analysis, PCP was mixed with potassium bromide at a ratio of 1:100 and compressed into a disk before applying on a Fourier transform infrared spectrophotometer (FT-IR, PerkinElmer Spectrum 100).

Lymphocyte proliferation assay

Mice splenocytes (2*105 cells/well) were cultured with PCP at 10, 50 and 250 µg/ml for 48 h in a 96-well plate. PMA/ionomycin and CpG were used as positive controls. The supernatants were collected for measuring IL-2 production with commercial ELISA kit (BioLegend, US). Cell proliferation were detected using CCK-8 assay as described.

**Reference**

[1] Shen, X.D.; Perreault, H. Characterization of carbohydrates using a combination of derivatization, high-performance liquid chromatography and mass spectrometry. J Chromatogr A 1998, 811, 47-59.
